# Differential Expression of Chemokine and Matrix Re-Modelling Genes Is Associated with Contrasting Schistosome-Induced Hepatopathology in Murine Models

**DOI:** 10.1371/journal.pntd.0001178

**Published:** 2011-06-07

**Authors:** Carly R. Perry, Melissa L. Burke, Deborah J. Stenzel, Donald P. McManus, Grant A. Ramm, Geoffrey N. Gobert

**Affiliations:** 1 Molecular Parasitology Laboratory, Queensland Institute of Medical Research, Herston, Brisbane, Australia; 2 Faculty of Science and Technology, Queensland University of Technology, Gardens Point Campus, Brisbane, Queensland, Australia; 3 Hepatic Fibrosis Group, Queensland Institute of Medical Research, Herston, Brisbane, Australia; University of Edinburgh, United Kingdom

## Abstract

The pathological outcomes of schistosomiasis are largely dependent on the molecular and cellular mechanisms of the host immune response. In this study, we investigated the contribution of variations in host gene expression to the contrasting hepatic pathology observed between two inbred mouse strains following *Schistosoma japonicum* infection. Whole genome microarray analysis was employed in conjunction with histological and immunohistochemical analysis to define and compare the hepatic gene expression profiles and cellular composition associated with the hepatopathology observed in *S. japonicum-*infected *BALB*/c and CBA mice. We show that the transcriptional profiles differ significantly between the two mouse strains with high statistical confidence. We identified specific genes correlating with the more severe pathology associated with CBA mice, as well as genes which may confer the milder degree of pathology associated with BALB/c mice. In BALB/c mice, neutrophil genes exhibited striking increases in expression, which coincided with the significantly greater accumulation of neutrophils at granulomatous regions seen in histological sections of hepatic tissue. In contrast, up-regulated expression of the eosinophil chemokine *CCL24* in CBA mice paralleled the cellular influx of eosinophils to the hepatic granulomas. Additionally, there was greater down-regulation of genes involved in metabolic processes in CBA mice, reflecting the more pronounced hepatic damage in these mice. Profibrotic genes showed similar levels of expression in both mouse strains, as did genes associated with Th1 and Th2 responses. However, imbalances in expression of matrix metalloproteinases (e.g. *MMP12, MMP13*) and tissue inhibitors of metalloproteinases (*TIMP1*) may contribute to the contrasting pathology observed in the two strains. Overall, these results provide a more complete picture of the molecular and cellular mechanisms which govern the pathological outcome of hepatic schistosomiasis. This improved understanding of the immunopathogenesis in the murine model schistosomiasis provides the basis for a better appreciation of the complexities associated with chronic human schistosomiasis.

## Author Summary

Schistosomiasis is a significant cause of morbidity and mortality in the tropical world although its true burden has been historically underestimated. Millions of people currently endure severe pathology as a result of schistosome infections, although some individuals appear to be less susceptible to infection despite constant parasite exposure. A similar range of disease susceptibility is evident in different strains of inbred mice infected with schistosomes, thereby mirroring the clinical situation. Granuloma formation in the liver of both humans and mice is a characteristic manifestation of chronic schistosomiasis, and is largely controlled by gene signalling pathways. Certain genes expressed in particular cohorts of mice and humans may be associated with the development of severe pathology, or may confer a protective phenotype. This murine study highlights some key molecular aspects of chronic schistosomiasis which may be responsible for the development of both mild and severe pathology, and provides a bench mark for studying the mechanisms of schistosome-induced disease in humans.

## Introduction

Schistosomiasis currently afflicts over 200 million people and continues to cause debilitating disease worldwide, particularly in developing and resource-poor countries [1] where the true global impact of the disease has been largely unacknowledged [2]. The scope of morbidity caused by schistosomiasis japonica ranges from relatively mild hypersensitivity reactions to severe hepatic and intestinal fibrosis, granuloma formation, hepatosplenomegaly and portal hypertension [3]. This variation in human pathology appears largely dependent on the host immune response to schistosomes, and more specifically to the intensity of infection, the number of previous infections and co-infections with other parasites [4]. Similar variations in pathology during progressive schistosomiasis are known to occur between different mouse strains [5], [6], [7], such as inbred CBA, BALB/c and C57BL/6 mice, which again are likely to be attributable to differences in the modulation of host immune responses as other aforementioned contributing factors are controlled.

Schistosome-induced granuloma formation is characterised by a focussed accumulation of immune cells and collagen deposition, all of which attempt to neutralise the presence of parasitic eggs. This response is a manifestation of the host CD4+ T-cell dependent immune response against schistosome eggs lodged in the liver characterised by the production of the Th2 cytokines IL-4 and IL-13 that induce granuloma formation and fibrosis [4] [8].

A recent study by Burke *et al.* [8] of the molecular and cellular mechanisms occurring in the murine host liver (C57BL/6 mice) during schistosome infection demonstrated that genes with specific biological functions, particularly cytokines and chemokines, are differentially expressed in correlation with disease development [8]. The present study builds on this previous report [8], and examines not only the initiation and progression of schistosome-induced disease but also the severity of the murine host response to *Schistosoma japonicum*. We hypothesised that the contrasting pathology seen in BALB/c and CBA mice is due to differing gene expression within the livers of the two mouse strains, and that relative gene transcription levels and their products are also associated with the severity of the host responses. We identified specific gene expression profiles and cell types associated with the development of both moderate and severe *S. japonicum*-induced pathology by examining the transcriptional, parasitological and histological features of the livers of these two mouse strains during an active *S. japonicum* infection. These data provide a basis for identification of new candidate molecules that may be targeted for the future development of novel anti-schistosome therapeutics and vaccines.

## Materials and Methods

### Ethics Statement

All work was conducted with the approval of the Animal Ethics Committee of the Queensland Institute of Medical Research (Project Number 288), which adheres to the Australian code of practice for the care and use of animals for scientific purposes, as well as the Queensland Animal Care and Protection Act 2001; Queensland Animal Care and Protection Regulation 2002.

### Mice and Parasites

Six to eight week old female BALB/c and CBA mice were anaesthetised and percutaneously infected with 12 *S. japonicum* cercariae (Mainland Chinese strain, Anhui population). Mice were sacrificed at 4 (n = 5 per strain), 7 (n = 5 per strain) and 9 weeks p.i. (n = 6 per strain), and the portal vein perfused to obtain adult worms. An additional four mice per strain were used as uninfected controls. Livers were collected from all mice, and individual lobes preserved in either formalin or RNAlater for histological analysis and RNA extraction, respectively. The number of adult worm pairs in each mouse was recorded, and the hepatic egg burden was evaluated by quantifying the number of eggs per gram (EPG) of liver as described [9]. Briefly, a weighed portion of liver was digested in 4% (w/v) potassium hydroxide to extract the eggs. Eggs were then resuspended in formalin, and the EPG were determined from the average number of eggs present in three 5 µl aliquots [8], [9].

### Histological Analysis

Formalin-fixed, paraffin embedded liver tissues from infected and control mice were sectioned (4 µm) and stained with Haematoxylin and Eosin (H&E) to determine granuloma area, and Sirius Red for collagen to measure progressive liver fibrosis [10]. Giemsa and Leder staining were performed to demonstrate eosinophil and neutrophil infiltration, respectively [8], [11], [12]. An Aperio Slide Scanner and Image Scope software were used to digitise and analyse light microscopy images (Aperio Technologies, Vista, USA). The percentage of granulomatous liver was determined using ImageJ 1.42 q software (National Institutes of Health, USA) by blind Point Counting Stereology (Aperio Technologies, Vista, USA) on H&E stained sections [13]. The distribution of schistosome eggs in each mouse liver was also assessed using the H&E-stained sections. The number of egg clusters per liver section was determined (X40magnification), where a cluster was defined as four or more eggs contacting each other. The average number of eggs in each cluster was also determined for each section. The percentage of collagen in the total liver were determined for each mouse using Aperio Technologies Positive Pixel Count (Aperio Technologies, Vista, USA) as described [14]. Neutrophils and eosinophils were semi-quantified by averaging the number of positively stained cells in 20 high power fields (X400 magnification).

### Isolation, Quality and Quantity of RNA

Total RNA was extracted from liver tissues as described [8], [15]. Briefly, each liver sample (∼100 mg) was homogenised in TRIZOL reagent (Invitrogen, Carlsbad, USA) using a Tissuelyser (Qiagen, Valencia, USA). A fraction of the homogenate was then processed by phase extraction with chloroform and column chromatography using an RNeasy Mini Kit (Qiagen, Valencia, USA). RNA was quantified using a Nanodrop-1000 spectrophotometer (Nanodrop Tech, Wilmington, USA) and quality assessed using an Agilent 2100 Bioanalyzer (Agilent Tech, Foster City, USA) on the basis of RNA Integrity Number (RIN). For both mouse strains, three biological replicates from each of the infected and control groups were selected on the basis of highest RIN, adequate RNA concentration, and similarity egg burden and hepatic pathology. Thus, a total of 24 individual biological replicates were selected for separate microarray analysis.

### Microarray Analysis

#### Complementary RNA synthesis and array hybridisation

Complementary RNA was synthesised from 1 µg of total RNA from each of the 24 selected samples using an Illumina TotalPrep RNA Amplification Kit (Ambion, Austin, USA). Purified, biotinylated-cRNA was hybridised to Illumina MouseWG-6 v2 arrays, which were then scanned and digitised using an Illumina BeadStation according to the manufacturer's instructions (Illumina, San Diego, USA). All raw microarray data have been submitted to NCBI's Gene Expression Omnibus and are publicly available, with series accession number GSE25713. Fold changes and standard deviations observed for all genes are summarised in Tables S1 and S2.

#### Data analysis

Quality control (GenomeStudio; Illumina, San Diego, USA) involved examining signal intensity histograms for experimental noise (biological/technical variation), background interference and hybridisation controls. Expression data were entered into GeneSpring GX version 11 (Agilent Tech, Foster City, USA), scaled to the median of all samples, and baselined to the mean of the appropriate control samples for either mouse strain. Normalised gene-lists were filtered for significant signal on the basis of detection score, a measure of signal intensity relative to background controls. For a given gene to be accepted for further analysis, at least half of all samples were required to have a detection score ≥0.95 for that gene (which equated to a confidence value of p≤0.05) [8]. Two-Way ANOVA (p≤0.05) with Benjamini-Hochberg correction for multiple testing was then used to identify genes whose expression changed significantly over time and differed significantly between the BALB/c and CBA mice. An arbitrary cut-off of ±2 fold change in expression over time or between strains in at least one individual time-point was applied [12], [16].

#### DAVID analysis

DAVID (Database for Annotation, Visualization and Integrated Discovery) analysis was used to identify biological functions and pathways that were over-represented by any differentially expressed genes [17]. From the filtered gene-lists for each strain and time-point, functional annotation charts were produced to identify significantly enriched gene ontology terms and associations within the KEGG and/or Biocarta pathways. Functional annotation clustering was performed to identify relationships between enriched ontologies, thereby enabling the identification of gene subsets associated with similar biological processes [8].

### Real-Time PCR

Real-time PCR was performed to validate the expression patterns of a subset of genes determined by the microarray analysis. Complementary DNA (cDNA) was synthesised from total RNA using a QuantiTect Reverse Transcription Kit (Qiagen, Valencia, USA). cDNA concentration was determined using Nanodrop-1000 spectrophotometry. Primers were sourced from previous studies [8], [18], [19], [20], [21] or were designed using Primer3 (http://biotools.umassmed.edu/bioapps/primer3_www.cgi) software (Table S3) [22]. Data for each sample were normalised to the housekeeping gene, hypoxanthine phosphoribosyltransferase [23]. Real-time PCR was performed using SYBR green master mix (Applied Biosystems, Warrington, UK) on a Rotor-Gene 6000 (Corbett Life Sciences, Concorde, Australia).

### Statistical Analysis

Statistical analysis of all parasitological, histological and real-time PCR data involved 2-Way ANOVA with Bonferroni post-hoc testing (p≤0.05) (GraphPad Prism 5.0, San Diego, USA). Correlations between the PCR and microarray data were assessed as previously reported [8], [24]. Briefly, the “D'Agostino & Pearson omnibus normality test” and “Shapiro–Wilk normality test” were used to assess the distribution of the data. As the data were not normally distributed, a Spearman's Rho correlation was employed as described [24], [25], [26].

## Results

### Parasitological and Histological Analysis

**Figure 1:**
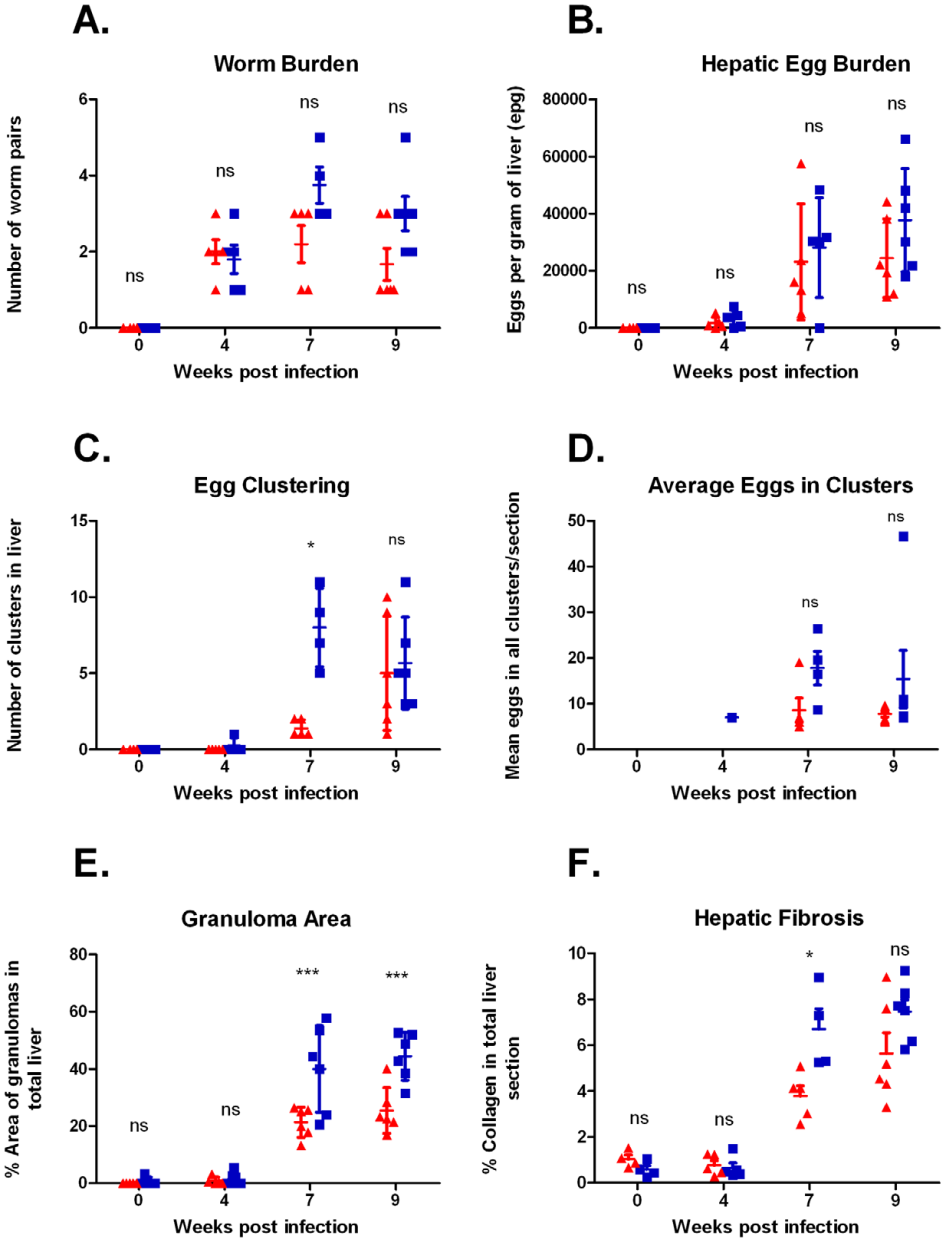
Parasitological and histological comparisons between BALB/c and CBA mice. Worm abundance (**A**) and hepatic egg burden (**B**) did not differ significantly between strains; egg clustering (**C**) was significantly greater in CBA mice compared to BALB/c mice at 7 weeks p.i, yet the mean number of eggs per cluster (**D**) did not differ significantly between strains. Granuloma area (**E**) was significantly greater in CBA mice compared to BALB/c mice, as was hepatic fibrosis at 7 weeks p.i (**F**). Statistical significance between strains was determined using 2-Way ANOVA with Bonferroni post hoc tests. ▴ = BALB/c replicates, ▪ = CBA replicates, *** = p<0.001, * = p<0.05, ns = no significant difference. Error bars represent mean with standard error of the mean (SEM).

There were no significant differences in worm burdens between strains or over time in infected mice (Figure 1A) (2-Way ANOVA, p>0.05). Further, no significant difference in hepatic egg burden was evident between the mouse strains at any time-point (Figure 1B) (2-Way ANOVA, p>0.05); however, hepatic egg burdens increased dramatically over time in both strains and was significantly higher at 7 and 9 weeks p.i. compared to 4 weeks p.i. (2-Way ANOVA, p<0.001). Egg distribution analysis indicated that a significantly higher number of egg clusters were present in CBA mice compared to BALB/c mice at 7 weeks p.i. (Figure 1C) (2-Way ANOVA, p<0.05); yet the mean number of eggs per cluster did not differ significantly between the mouse strains (Figure 1D) (2-Way ANOVA, p>0.05).

**Figure 2:**
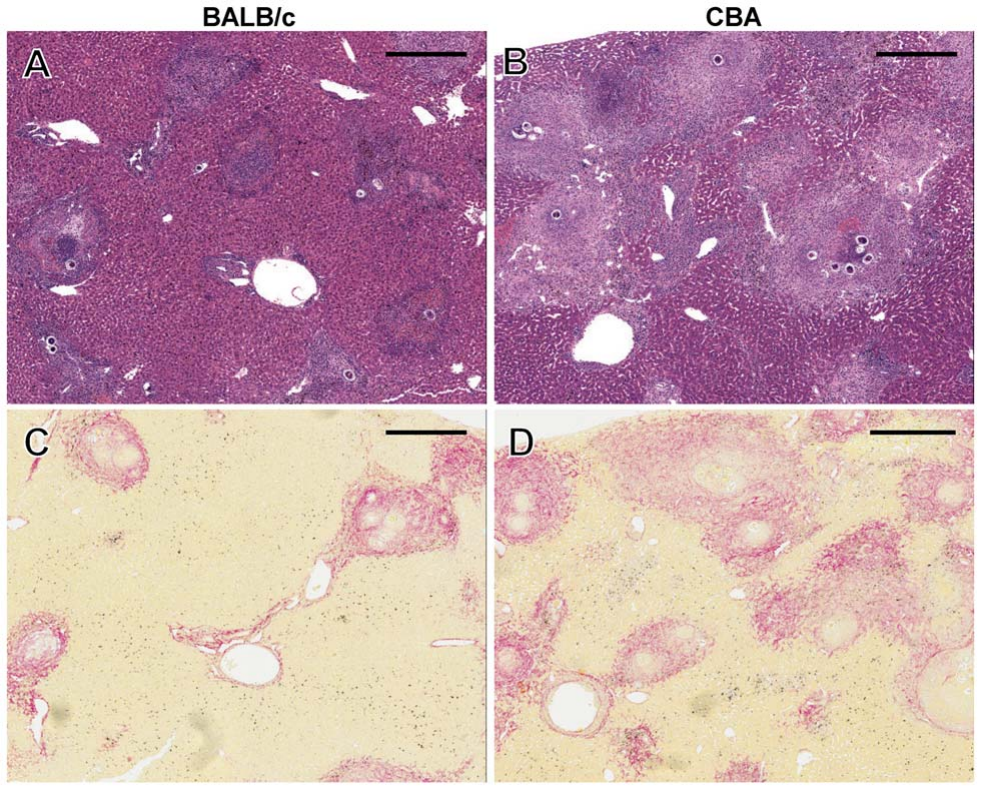
Histological staining highlights differences in tissue damage between BALB/c and CBA mice. Granulomatous pathology was more severe in CBA mice despite a similar egg burden (**A** and **B**; Haematoxylin and Eosin x40). Collagen deposition (red) was also greater in CBA mice (**C** and **D**; Sirius Red x40). All images were derived from mice at 7 weeks p.i. and were taken from murine livers with similar egg burdens. **A** and **C**; **B** and **D** were taken from sections of the same mouse. Scale bar equals 400 µm.

Granuloma area was significantly greater in the CBA mice compared to the BALB/c mice. In CBA mice, the granuloma area represented 43.8% and 44.4% of the total liver area at 7 and 9 weeks p.i. respectively, whereas the BALB/c granuloma area was, respectively, 22.1% and 25.4% of the total liver area (Figures 1E, 2A and 2B) (2-Way ANOVA, p<0.001).

Hepatic fibrosis was induced more rapidly in the CBA mice compared to the BALB/c mice. This was reflected by significantly greater collagen deposition in the CBA mice at 7 weeks p.i., where collagen represented 6.7% of the total liver area, compared to 3.8% in BALB/c mice (Figures 1F, 2C and 2D) (2-Way ANOVA, p<0.05). Collagen deposition was comparable between the two strains at 9 weeks p.i., indicating that the BALB/c fibrotic response develops further over time to parallel that observed in CBA mice.

**Figure 3:**
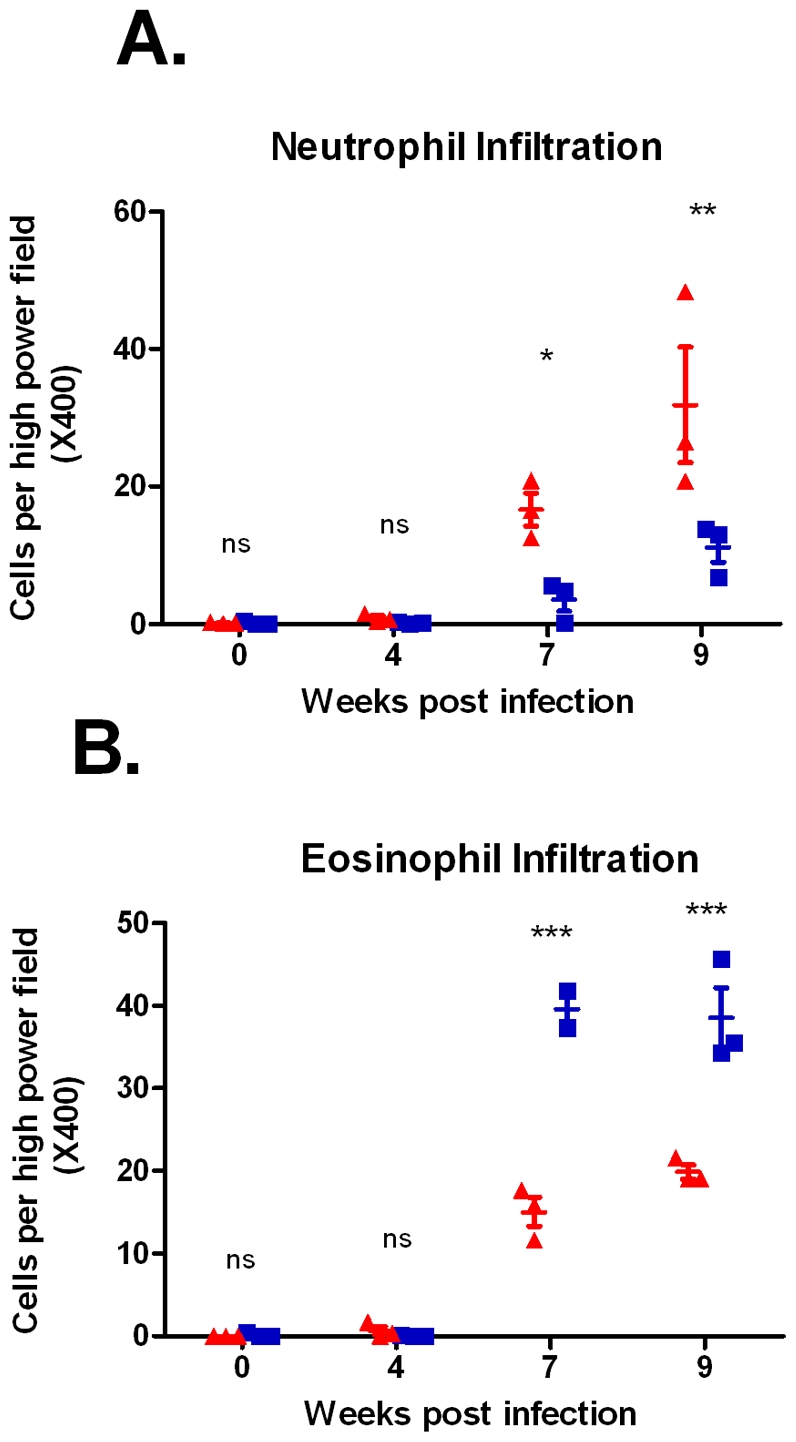
Comparison of cell infiltration in CBA mice and BALB/c mice. Hepatic neutrophil infiltration (**A**) was markedly higher in BALB/c mice compared to CBA mice. Conversely, eosinophil numbers (**B**) were significantly greater in CBA mice compared to BALB/c mice. Statistical significance between strains was determined using 2-Way ANOVA with Bonferroni post hoc tests. ▴ = BALB/c replicates, ▪ = CBA replicates, *** = p<0.001, ** = p<0.01, * = p<0.05, ns = no significant difference. Error bars represent mean with standard error of the mean (SEM).

**Figure 4:**
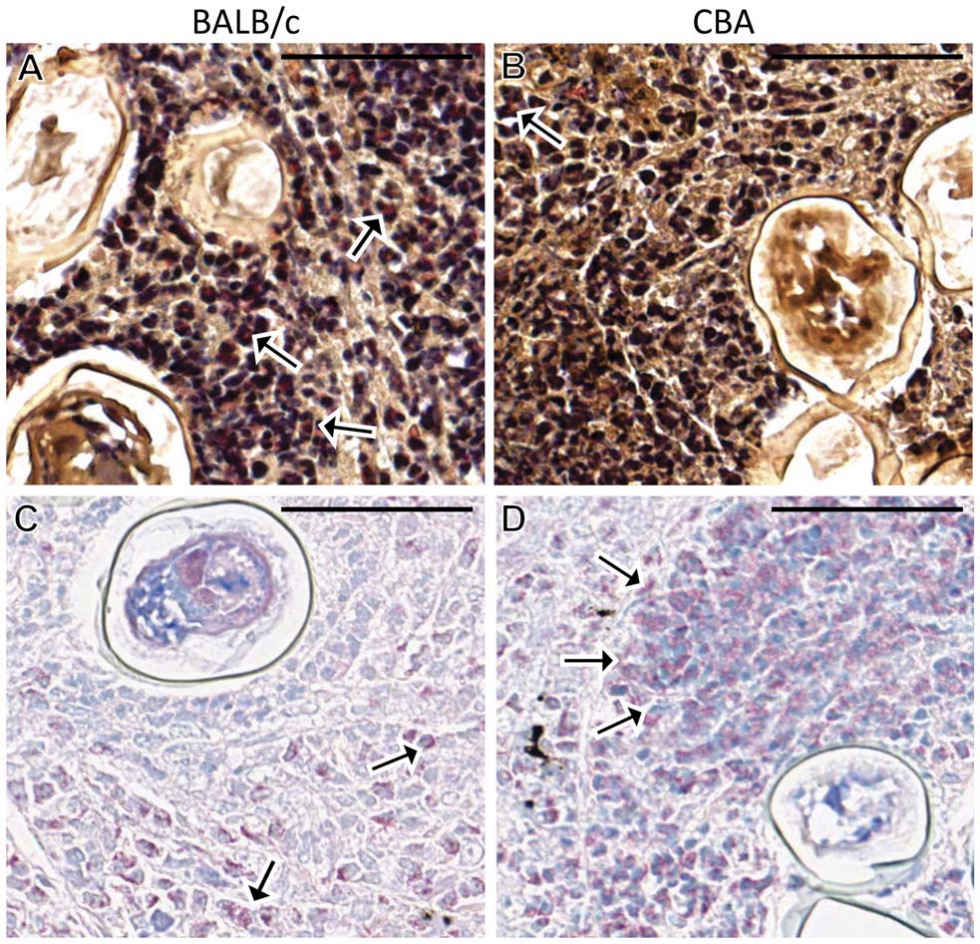
Cellular infiltration differs between BALB/c and CBA mice. Large areas of neutrophil accumulation (Leder stain; cells with pink cytoplasm indicated by arrows) adjacent to eggs were more common in BALB/c mice (**A**) compared with relatively scarce neutrophil accumulation in the granulomas of CBA mice (**B**). Conversely, large areas of eosinophil accumulation (Giemsa stain; cells with pink cytoplasms indicated by arrows) were more frequent in the granulomas of CBA mice (**D**) compared with those in BALB/c mice (**C**). All images were derived from mice at 7 weeks p.i. and were taken from murine livers with similar egg burdens and represent granulomas containing a similar number of eggs and at a similar stage of development. Scale bar = 50 µm (X400).

Leder staining indicated that neutrophil infiltration to the liver was significantly higher in the BALB/c mice at both 7 and 9 weeks p.i., with means of 16.6 and 31.9 neutrophils per high power field (/hpf) at these respective time-points compared to means of 3.6 and 11.2 neutrophils/hpf in the CBA mice (Figures 3A, 4A and 4B) (2-Way ANOVA, p<0.05).

In contrast, Giemsa staining indicated that the eosinophilic response was significantly higher in CBA mice (Figures 3B, 4C and 4D) (2-Way ANOVA, p<0.001). Means of 39.6 and 38.5 eosinophils/hpf were present in the CBA livers at 7 and 9 weeks p.i. respectively, compared to means of 15.0 and 19.9 eosinophils/hpf in BALB/c mice at these same time-points.

### Microarray Analysis

#### Data normalisation and filtration

**Figure 5:**
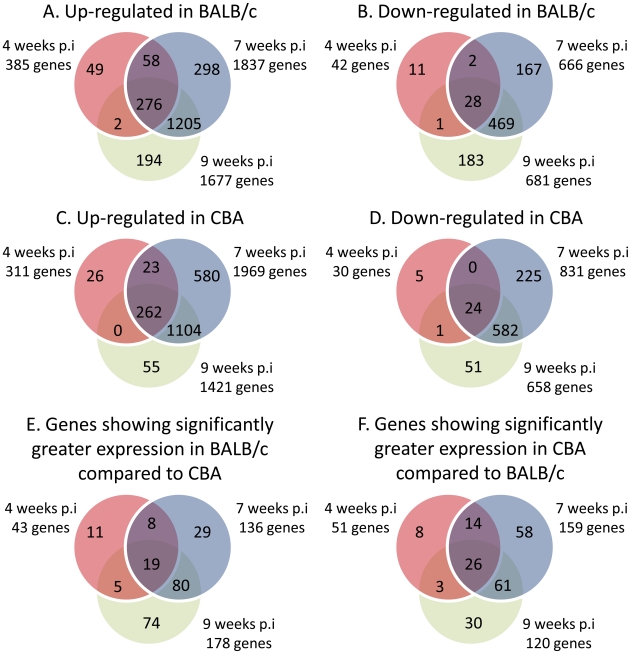
Venn diagrams to illustrate the overlap between genes showing significantly altered expression. **A** and **B** are derived from the list of 2,937 genes which were significantly changing over time in BALB/c mice (2-Way ANOVA, p<0.05). **A** shows the overlapping expression of genes within this 2,937 gene list that were significantly up-regulated at either 4, 7 or 9 weeks p.i. **B** demonstrates the overlap of expression for genes which were down-regulated at either 4, 7 or 9 weeks p.i. Similarly, **C** and **D** correspond to the 2,938 genes showing differential expression over time in CBA mice (2-Way ANOVA, p<0.05), where **C** represents up-regulated genes and **D** represents down-regulated genes. The majority of these genes show overlapping expression between 7 and 9 weeks p.i. **E** and **F** represent the 687 genes which were differentially expressed between strains (2-Way ANOVA, p<0.05). **E** depicts the overlapping expression of genes which were more highly expressed in BALB/c mice at either 4, 7 or 9 weeks p.i. **F** demonstrates the overlapping time-points at which genes transcribed at much higher levels in CBA mice were expressed.

Filtering of normalised expression data on the basis of detection score reduced the data set from 45,281 to 18,243 genes. Next, 2-Way ANOVA was applied, resulting in the identification of 8,937 differentially expressed genes over time in either BALB/c or CBA mice (2-Way ANOVA, p≤0.05). These gene lists were further refined to identify changes in expression with likely biological significance by applying a ±2-fold cut-off in at least one time-point for up- and down-regulated genes. In the CBA mice, 2,938 genes exhibited at least a 2-fold change in expression over time, as did 2,937 genes in the BALB/c mice. These gene lists were further subdivided into lists of genes showing ≥2-fold up-regulation or ≥2-fold down-regulation for at least one individual time-point. Notably, 1,733 genes were identified whose expression differed significantly between the two mouse strains (2-Way ANOVA, p≤0.05; Table S4). Of these, 687 genes showed at least ±2-fold differential expression. This list was then subdivided into lists of genes showing greater expression in either BALB/c or CBA mice at each time-point. The overlap between these lists throughout the time-course of infection and between the two strains is shown in Figure 5.

#### Functional annotation analysis

Gene-lists were analysed by functional annotation clustering using DAVID. This enabled the identification of common biological clusters or themes associated with progression of infection in both mouse strains (Tables S5 and S6) and differences in key biological themes that may have contributed to the observed differences in pathology.

#### Up-regulated genes are associated primarily with inflammation and immunology

*General pattern of expression:* Genes that were up-regulated over the course of *S. japonicum* infection were associated with immune system processes, inflammatory responses, antigen processing and presentation, T-cell activation, cell migration, chemokine and cytokine activity and wound healing. This was a common expression pattern for both the BALB/c and CBA mice.

*Th1 and Th2-associated gene expression is similar in both mouse strains:* Key cytokines associated with polarised Th1/Th2 responses showed no significant difference in expression between the two mouse strains (Table 1). Hepatic expression of Th1 associated genes Interleukin-1α (*IL-1α*), Interferon-γ Receptor 1 (*IFN-γR1*), Tumour Necrosis Factor-α (*TNF-α*) and Signal Transducers and Activators of Transcription 1 (*STAT1*) was significantly up-regulated over time in both BALB/c and CBA mice (1-Way ANOVA, p<0.05). *STAT1* showed an expression pattern typical of the early schistosome-induced Th1 response, peaking at 4 weeks p.i. and declining thereafter in both strains. Expression of *IL-1α* and *TNF-α* peaked at 7 weeks p.i. in both strains, whereas *IFN-γR1* expression peaked at 9 weeks p.i. in BALB/c mice and at 7 weeks p.i. in CBA mice. Expression of Th2-associated profibrotic cytokines Interleukin-10 Receptor Alpha (*IL-10Rα*), Interleukin-4 (*IL-4*), Interleukin-13 (*IL-13*), Interleukin-33 (IL-33) and Interluekin-4-inducible 1 (Il4i1) was significantly increased over time in both mouse strains (1-Way ANOVA, p<0.05) but did not differ significantly between strains (2-Way ANOVA p>0.05). *IL-4* expression peaked at 9 weeks p.i. in BALB/c mice, and at 7 weeks p.i. in CBA mice; *IL-13* showed peak expression in BALB/c mice at 7 weeks p.i. and in CBA mice at 9 weeks p.i. Other Th1/Th2 associated genes such as IFNγ, IL-12, IL-12R and IL-4R were unmeasurable by microarray analysis (i.e did not exhibit significant signal relative to background).

**Table 1:** Microarray expression patterns for genes of interest in BALB/c and CBA mice.

	Expression fold-change* relative to uninfected mice						
	BALB/c			CBA			
Gene Description	4 wks	7 wks	9 wks	4 wks	7 wks	9 wks	Probe ID
***Up-regualted genes***							
Pro-Platelet Basic Protein (PPBP)	4.2	20.9	15.2	1.0	4.4	2.5	ILMN_2908435 ILMN_1228102
Cathepsin G (CTSG)	1.9	78.3	82.7	1.1	26.2	14.2	ILMN_1220236
Neutrophilic Granule Protein (NGP)	2.2	136.0	171.0	1.1	50.7	24.1	ILMN_1228832
Myeloperoxidase (MPO)	3.1	109.7	121.4	1.2	52.3	26.9	ILMN_2925094 ILMN_2719256 ILMN_2600421 ILMN_1249030
S100 Calcium Binding Protein A8 (S100A8)	3.1	67.0	65.9	2.0	191.0	164.8	ILMN_2710905
Chemokine (C-C motif) Ligand 24, Eotaxin-2 (CCL24)	1.8	2.1	2.2	5.2	15.8	7.7	ILMN_1225406
Eosinophil-Associated Ribonuclease A, Family Member 11 (EAR11)	1.5	13.9	54.6	1.6	160.8	115.7	ILMN_2890019
Mannose Receptor C, Type 1 (MRC1)	1.3	1.6	1.2	1.4	3.3	2.3	ILMN_1239430
Chitinase 3-Like 3 (CHI3L3)	1.3	40.6	66.3	1.3	111.3	74.8	ILMN_3117876
Resistin like alpha (RETNLA)	1.0	4.6	25.5	1.4	130.9	111.9	ILMN_1226472
***Down-regulated genes***							
Cytochrome P450, Family 2, Subfamily A, Polypeptide 4 (CYP2A4)	−1.3	−5.7	−6.3	−1.6	−45.0	−39.3	ILMN_1250364
Cytochrome P450, Family 2, Subfamily B, Polypeptide 10 (CYP2B10)	−1.8	−8.7	−16.6	−1.5	−41.0	−40.0	ILMN_2594926
Cytochrome P450, Family 2, Subfamily B, Polypeptide 23 (CYP2B23)	−1.9	−9.1	−11.0	−1.4	−54.8	−53.1	ILMN_2976211
***Fibrotic genes***							
Collagen Type 1, Alpha 1 (COL1A1)	1.7	36.3	31.6	1.3	40.0	21.0	ILMN_2687872
Chemokine (C-X-C motif) Ligand 1 (CXCL1)	−1.4	4.3	4.2	1.3	4.7	5.8	ILMN_2763245
Tissue Inhibitor of Metalloproteinase 1 (TIMP1)	1.4	52.9	43.6	1.5	109.2	60.3	ILMN_2769918 ILMN_3103896
Matrix Metallopeptidase-12 (MMP12)	1.3	5.0	4.8	1.4	47.7	28.8	ILMN_1250421
Matrix Metallopeptidase-13 (MMP13)	2.8	4.6	1.9	2.0	57.1	21.9	ILMN_2737685
Platelet-Derived Growth Factor-Beta (PDGF-β)	1.4	2.7	2.3	1.5	3.7	2.4	ILMN_2618714
Transforming Growth Factor-Beta 1 (TGF-β1)	1.5	3.6	3.0	1.2	2.9	2.0	ILMN_2711461
Connective Tissue Growth Factor (CTGF)	1.1	1.8	1.9	1.1	2.2	1.9	ILMN_2711461 ILMN_2909150
Actin Alpha-2 Smooth Muscle Aorta (ACTA2)	−1.2	1.8	1.9	1.2	2.0	2.0	ILMN_2710353 ILMN_2710354 ILMN_2693895 ILMN_2923445
***Th1 genes***							
Interferon-Gamma Receptor 1 (IFN-γr1)	1.4	2.5	2.7	1.5	2.9	2.5	ILMN_2651575
Tumour Necrosis Factor-Alpha (TNF-α)	1.7	2.8	2.3	1.5	2.0	1.4	ILMN_2899863
Interleukin-1 Alpha (IL-1α)	1.7	3.6	1.9	1.8	2.3	1.5	ILMN_1243066 ILMN_1227018
Signal Transducers and Activators of Transcription 1 (STAT1)	3.6	2.3	1.7	2.3	1.9	1.2	ILMN_2655721 ILMN_2593196 ILMN_2510233
***Th2 genes***							
Interleukin-4 (IL-4)	1.4	2.5	2.7	1.4	4.1	2.9	ILMN_2931334
Interleukin-10 Receptor Alpha (IL-10rα)	2.5	3.9	2.6	1.4	2.3	1.7	ILMN_1219946
Interleukin-13 (IL-13)	1.1	1.5	1.1	1.2	1.4	1.8	ILMN_2927131
Interleukin-33 (IL-33)	−1.1	3.0	2.6	−1.0	5.3	3.7	ILMN_1259747
Interleukin-4 inducible 1 (IL4i1)	1.8	2.3	1.9	1.9	3.6	2.2	ILMN_2733778

*Expression is presented as fold-change relative to uninfected controls for each strain. Negative values represent down-regulation. Fold changes were averaged for genes represented by two or more probes.

*Genes differentially expressed in BALB/c and CBA mice:* The majority of differentially up-regulated genes between strains occurred at the 7 and 9 week time-points, and coincided with the greatest difference in hepatic granulofibrosis between strains (Figure 1C and 1F). The specific fold changes of genes referred to in this and subsequent sections are summarised in Table 1.

Up-regulated genes showing differential expression between the two strains were predominantly associated with biological clusters including “Immune Response”, “Chemokine and Cytokine Activity”, “Inflammatory and Wound Response”, “Cell Activation” and “Cell Migration and Locomotion” (Table 1). Prominent genes detected within these clusters were specifically associated with neutrophils, eosinophils, macrophages and fibrosis.

Expression of Pro-Platelet Basic Protein (*PPBP* or *CXCL7*), a chemokine that promotes neutrophil adhesion and transendothelial migration [27], was significantly higher in BALB/c mice at 4, 7 and 9 weeks p.i. compared to CBA mice (Table 1) (2-Way ANOVA, p<0.05). The greatest difference in *PPBP* expression between strains occurred at 9 weeks p.i., where the livers of BALB/c mice transcribed approximately 6 times more.

Cathepsin G (*CTSG*), a proteolytic constituent of neutrophilic granules [28], showed significantly higher gene expression in BALB/c mice at 7 and 9 weeks p.i. reaching levels approximately 6 times that observed in CBA mice (Table 1) (2-Way ANOVA, p<0.05).

Other genes indicative of neutrophil infiltration, including Neutrophilic Granule Protein (*NGP*) and Myeloperoxidase (*MPO*) increased significantly in both mouse strains over time (1-Way ANOVA, p<0.05), but this increase was significantly greater in BALB/c mice compared to CBA mice (Table 1) (2-Way ANOVA, p<0.05). Up-regulation of *NGP* and *MPO* was sustained in BALB/c mice over the time-course, whereas the expression of these genes peaked at 7 weeks p.i. in CBA mice and then declined. *S100A8*, a chemotactic molecule for neutrophils [29], was significantly up-regulated in both mouse strains over time (1-Way ANOVA, p<0.05). However, CBA mice showed a significantly greater increase in expression of *S100A8* compared to BALB/c mice at 7 and 9 weeks p.i. (Table 1.) (2-Way ANOVA, p<0.05).

Expression of *CCL24*, a chemokine that induces chemotaxis in eosinophils [30], differed significantly between BALB/c and CBA mice (Table 1) (2-Way ANOVA, p<0.05). *CCL24* was significantly up-regulated in CBA mice at 4, 7 and 9 weeks p.i. (1-Way ANOVA, p<0.05), but not at any time-point in BALB/c mice. Eosinophil-associated ribonuclease A family member 11 (*EAR11*), an additional marker of eosinophil infiltration, also showed significantly higher expression in CBA mice compared to BALB/c mice (Table 1) (2-Way ANOVA, p<0.05). *EAR11* expression was approximately 11 times greater in CBA mice at 7 weeks p.i.

Chitinase 3-Like 3 (*CHI3L3*), Resistin like alpha (*RETNLA*) and Mannose Receptor C Type 1 (*MRC1*), markers for alternatively activated macrophages (AAMs), also exhibited differential expression between the two strains. Expression of these genes was significantly greater in CBA mice (Table 1) (2-Way ANOVA, p<0.05), particularly at 7 weeks p.i. where the expression of all three genes was more than twice that expressed in BALB/c mice.

#### Genes involved in fibrosis

As hepatic fibrosis was significantly greater in hepatic sections from CBA than from BALB/c mice, genes specifically associated with this process were examined. Genes associated with fibrosis and related ontologies showed significant up-regulation in both mouse strains throughout the time-course, although only a subset showed differential expression between strains (Table 1).

Collagen Type 1, Alpha 1 (*COL1A1*) and the hepatic stellate cell chemokine *CXCL1* were significantly up-regulated at 7 and 9 weeks p.i. in both BALB/c and CBA mice (1-Way ANOVA, p<0.05). There was no significant difference in *COL1A1* expression between strains (Table 1) (2-Way ANOVA, p>0.05), and *CXCL1* was differentially expressed only at 4 weeks p.i. (Table 1) (2-Way ANOVA, p<0.05).

Fibrosis-associated genes Tissue Inhibitor of Metalloproteinase 1 (*TIMP1*) and Matrix Metalloproteinases 12 and 13 (*MMP12*, *MMP13*) were significantly up-regulated in both BALB/c and CBA mice at 7 and 9 weeks p.i. (Table 1) (1-Way ANOVA, p<0.05). *TIMP1* expression differed significantly between strains, with CBA mice showing much greater fold-changes than BALB/c mice (2-Way ANOVA, p<0.05). Similarly, expression of *MMP*s *12* and *13* was up-regulated to a significantly greater extent in CBA mice (2-Way ANOVA, p<0.05).

Profibrotic cytokines Platelet-Derived Growth Factor-β (*PDGF-β*), Transforming Growth Factor-β1 (*TGF-β1*) and Connective Tissue Growth Factor (*CTGF*) showed no significant differences in expression between BALB/c and CBA mice throughout the time-course (Table 1) (2-Way ANOVA, p>0.05). The hepatic stellate cell marker Actin Alpha 2, Smooth Muscle (*α-SMA*) showed differential expression between the two strains only at 4 weeks p.i. (Table 1) (2-Way ANOVA, p<0.05).

#### Down-regulated genes are involved predominantly in metabolic processes

Genes that were down-regulated in both strains were assigned to clusters representing “Oxido-reductase Activity”, “Ion and Vitamin Binding”, “Biosynthetic Processes” and “Metabolic Processes”, including lipid, fatty acid and amine metabolism (Table S4). Notably, confluent cytochrome down-regulation was a common expression pattern across both *S. japonicum*-infected mouse strains (Table 1). Of the 71 cytochrome genes that were significantly down-regulated in either strain over the time-course (1-Way ANOVA, p<0.05), expression patterns for 4 genes were shown to differ significantly between strains. Cytochromes 2a4 (*CYP2A4*), 2b10 and 2b23 were significantly more down-regulated in CBA mice over the course of infection (Table 1) (2-Way ANOVA, p<0.05).

### Genbank Accession Numbers

*ACTA2* (*α-SMA)*: NM_007392.2; *CCL11*: NM_011330.1*; CCL24*: NM_019577.4; *CHI3L3*: NM_009892.1; *COL1A1*: NM_007742.2; *CTGF*: NM_010217.1; *CTSG*: NM_007800.1; *CXCL1*: NM_008176.1; *CYP2A4*: NM_009997.2; *CYP2A5*: NM_007812.2; *CYP*2*B10*: NM_009999.3; *CYP2B23*: NM_001081148.1; *EAR11*: NM_053113.2; *HPRT*: NM_013556.2.; *IFNγ*: NM_008337.3; *IFN-γR1*: NM_010511.2; *IL-10Rα*: NM_008348.2; *IL-12Rβ1*: NM_008353.1IL12Rβ2: NM_008354.3; *IL-12α*: NM_008351.1; *IL-12β*: NM_008352.2 *IL-13*: NM_008355.1; *IL-1α*: NM_010554.4; *IL-4*: NM_021283.1; IL-4Rα: NM_001008700.3; *MMP12*: NM_008605.3; *MMP13*: NM_008607.1; *MPO*: NM_010824.1; *MRC1*: NM_008625.1; *NGP*: NM_008694.1; NM_008353.1; *PDGF-β*: NM_011057.2; *PPBP (CXCL7)*: NM_023785.1 and NM_023785.2; *S100A8*: NM_013650.2; *S100A9*: NM_009114.1; *STAT1*: NM_009283.3; *TGF-β1*: NM_011577.1; *TIMP1*: NM_011593.2; *TNF-α:* NM_013693.1 and NM_013693.2.

### Real-Time PCR

**Figure 6:**
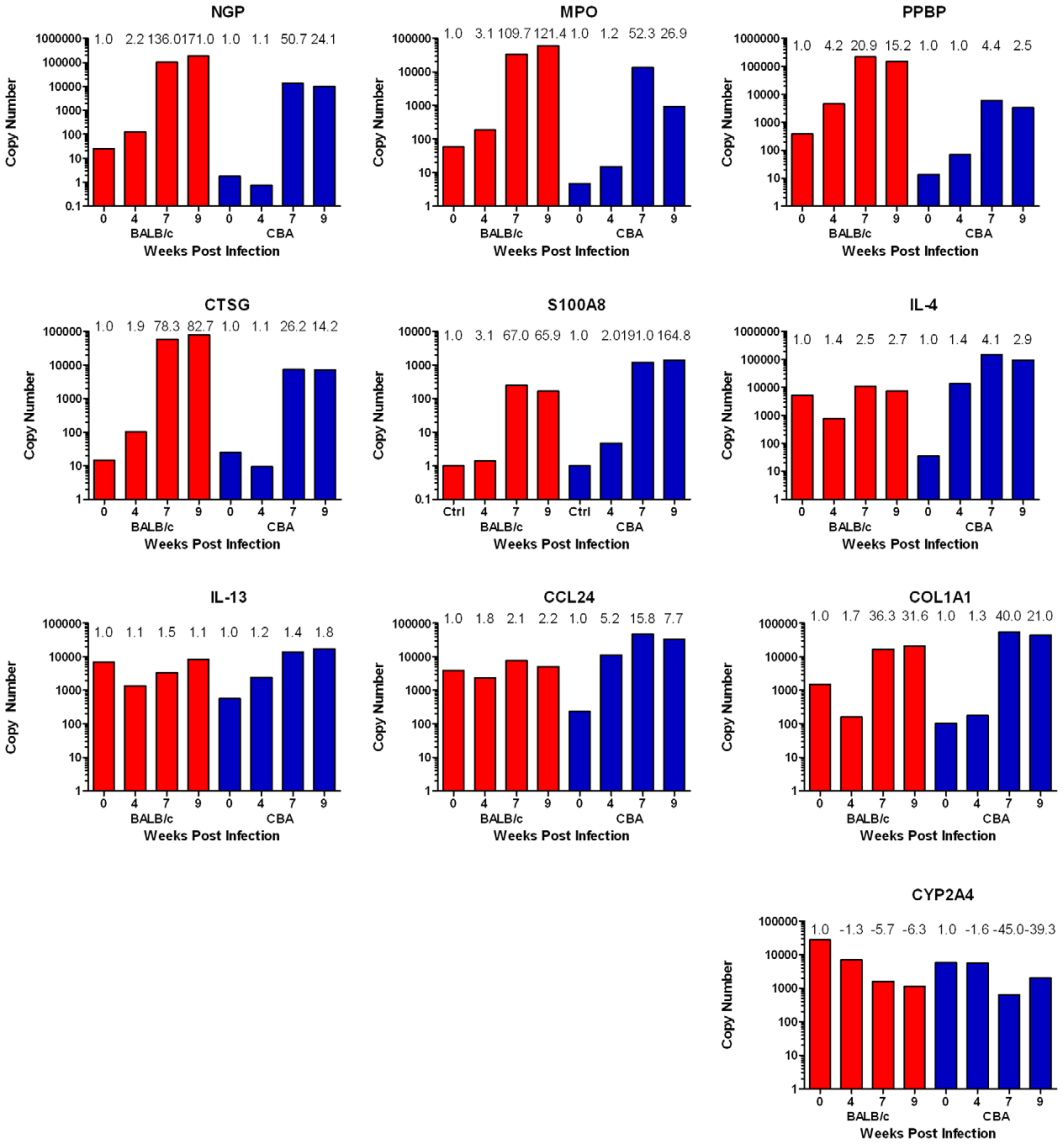
Real-time PCR validates microarray results. Microarray fold-changes relative to uninfected controls for each strain and time-point are presented above the individual bars. Real-time PCR data are presented in the column graphs as copy number. PCR copy number provides an absolute comparison between all groups; microarray fold-change provides a relative comparison between all groups, as these data are normalised to respective control groups.

Real-time PCR validated the expression patterns of a subset of genes (*CCL24*, *COL1A1*, *CTSG*, *CYP2A4*, *IL-4*, *IL-13*, *MPO*, *NGP*, *PPBP* and *S100A8*) selected from the microarray analysis (Figure 6). There was a significant correlation between the expression levels revealed by the real-time PCR and the microarray analysis (Spearman's Correlation; r = 0.62; p<0.001).

## Discussion

It is well documented that BALB/c and CBA mice show variation in susceptibility to infection and pathology associated with a number of pathogens including *Leishmania major, Streptococcus pneumonia,* the cause of acute pneumococcal disease [31], [32], and *Candida albicans* [33]. The pathological differences evident between these two mouse strains during these infections are attributable to variations in the levels of particular components of the host immune system [31], [32], [33].

Numerous studies have also demonstrated differing pathological outcomes in schistosome-infected BALB/c, CBA and C57BL/6 mice. C57BL/6 mice show higher susceptibility to *S. mansoni* infection than BALB/c mice [5], whereas BALB/c and C57BL/6 mice show less susceptibility to *S. japonicum* infection than CBA mice [7]. Furthermore, Hirata *et al.* showed that during a *S. japonicum* infection, inflammation ceased earlier in BALB/c mice, and CBA mice showed a greater degree of inflammatory and granulofibrotic responses [34]. However, the immunological mechanisms underlying these differences are yet to be fully realised.

The transcriptomic and histological data presented here provide a more complete picture of the molecular and cellular mechanisms which govern hepatic pathology in BALB/c and CBA mice. Our results confirm that the transcriptional profiles of BALB/c and CBA mice differ significantly during *S. japonicum* infection, and these differences may contribute to the development of either moderate or severe granulofibrotic pathology. Notably, we have identified for the first time particular gene subsets and cell populations which correlate with either severe or moderate *S. japonicum*-induced hepatopathology.

Non-significant differences in worm and hepatic egg burden between BALB/c and CBA mice indicates that severe pathology during schistosomiasis is not simply the result of more schistosome eggs producing more antigens. However, a significant difference in early egg distribution within hepatic granulomas of the BALB/c and CBA mice may reflect variability in the arrangement of eggs as they migrate to the liver from the mesenteric veins of the intestine, where *S. japonicum* eggs are characteristically deposited in clusters [35]. The immunological make-up of the two mouse strains may influence the ability of *S. japonicum* eggs to stay in close association during migration, and/or their capacity to re-cluster or remain clustered once they reach the liver. The clustered egg distribution of granulomas in CBA mice at 7 weeks p.i. coincided with a vastly greater percentage of granulomatous tissue compared to the BALB/c mice. Thus, it is possible that CBA granuloma area is significantly greater because the egg clustering makes them spatially larger, or the accumulation of localised egg antigens tips the threshold of a normal immune response such that more cells or different cell types are involved. It may also be that antigens associated with singly distributed or smaller clusters of eggs in the BALB/c liver could be neutralised more easily than those from larger egg clusters, where the inner egg mass may be inaccessible to immune effector cells; this is an important area for further investigation.

While specific genes involved in the CBA immune response are likely to be causing a greater degree of granulofibrotic pathology, it is also possible that the greater degree of pathology, caused by mechanisms independent of the immune response including antigen exposure, alters gene expression and the nature of the immune response itself. To address this issue of circular cause and consequence, fold-changes in gene expression were compared to the changes in hepatic granuloma area. Granuloma area was maximally 2.5-fold greater in CBA mice than in BALB/c mice, so we hypothesised that genes showing differences in gene expression higher than this were likely to be the cause of granulofibrotic pathology, rather than the consequence. Further, to control for inherent differences in the baseline gene expression between uninfected BALB/c and CBA mice we examined the level of induction (i.e fold change) of genes in infected CBA and BALB/c mice compared with uninfected mice of the same strain.

In both the BALB/c and CBA mice, the up-regulation of genes paralleled biological functions and cellular activities involved in granuloma formation and fibrosis and were consistent with other time-course studies of murine infection with *S. japonicum* [6], [8], [12], [36]. Many genes encoding cytokines, chemokines, enzymes or proteins were differentially up-regulated between the two mouse strains, and cell types quantified in histological sections mirrored these gene expression patterns. The greater burden of severe tissue damage in CBA mice compared to BALB/c mice was associated with considerably greater down-regulation of numerous cytochrome and metabolic genes (Table 1), a pattern consistent with other murine studies on *S. japonicum* [8] and *S. mansoni* [37]. Notably, the expression levels of Th1- and Th2-associated genes were similar in BALB/c and CBA mice, suggesting that factors other than the Th1/Th2 cytokine balance contribute to the difference in pathology between the two strains.

Our data indicate that the immune response in the lower pathology BALB/c mice involves a considerable influx of neutrophils, as reflected in the higher expression of the neutrophil markers *NGP*, *MPO* and *CTSG,* the neutrophil chemokine *PPBP* (*CXCL7*) and direct histological analysis. The role of neutrophils during *S. japonicum* infection is currently unknown, although our observations suggest they may play a regulatory role during the development of the resulting granulomatous pathology. That the fibrotic response of BALB/c mice parallels that in CBA mice by 9 weeks p.i. suggests that neutrophils are only effective in this regulatory capacity early in infection and later become inactivated or overwhelmed by the extent of resulting pathology. This proposed role for neutrophils is in direct contrast to models of *S. mansoni* infection in which neutrophils are thought to play only a minor role in granuloma development [38], [39]. Similar roles for neutrophils have been reported for a rat model of cholestatic liver disease in which neutrophils are essential for collagen reabsorption and tissue repair/remodelling [40] and other inflammatory responses, such as wound healing, in which neutrophilic influx correlates inversely with fibrosis [41]. The regulatory activity of neutrophils may be attributable to constituents of the neutrophilic granule proteins such as collagenase and gelatinase (i.e. MMP's), which degrade matrix proteins [42]. We hypothesise that the observed up-regulation and higher induction of *NGP*, *MPO* and *CTSG* in BALB/c mice in our study may confer similar activity. CTSG attacks collagen and fibronectin and inhibits TIMPs [43] while NGP and MPO are known to participate in immune defence mechanisms against microorganisms by releasing hydrolytic enzymes or toxic free radicals [44]. Thus, neutrophilic granule proteins could act to neutralise schistosome antigens, and to reduce fibrosis by attacking collagen and fibrin. This mode of action may confer protective characteristics in BALB/c mice, which may account for the less severe pathology observed with this strain compared to CBA mice. Conversely, it has been proposed that neutrophil CTSG acts to promote fibrin formation [45]. Thus, the role of CTSG remains ambiguous and requires further investigation. Future work to further clarify the role of neutrophils should include integrin activation and cell adhesion studies, to assess the specific anchoring of neutrophils within the liver. The use of knockout mouse models may define the specific contribution of the relevant genes and their products, including *MPO*, *NGP*, *CTSG* and *PPBP*.

In contrast to other neutrophil associated genes, S100A8 exhibited significantly higher induction in the higher pathology CBA mice compared with lower pathology BALB/c mice. S100A8 has previously been localised to regions of neutrophil accumulation during *S. japonicum* infection [8] and is proposed to play an important role in dictating the cellular composition of *S. japonicum*-induced granulomas [46], possibly by inducing chemotaxis and adhesion in neutrophils [29]. Conversely, high levels of S100A8 and the related molecule S100A9 are released from necrotic cells and can undergo oxidative modifications such that at high concentrations they act to limit inflammation and cellular recruitment [47]. Thus, while the relatively lower levels of S100A8 observed in BALB/c mice are likely to contribute to neutrophil recruitment and granuloma formation, the higher levels in CBA may reflect the higher degree of tissue damage observed in these mice and represent a last ditch compensatory mechanisms to limit excessive pathology [47].

Marked eosinophil accumulation and higher expression of the eosinophil-associated gene *EAR11* and chemokine *CCL24* (Eotaxin-2) in granulomas was a striking feature in the higher pathology CBA mice compared with BALB/c mice. It is well documented that eosinophils are intimately involved in inflammatory responses and defence mechanisms against parasites including *S. mansoni* during granuloma formation [30], [48]. An eosinophilic profile has also been reported in other chronic human liver diseases as well as asthma, atherosclerosis and pulmonary fibrosis, where the eosinophilic chemokine CCL11 (Eotaxin-1) has been implicated in pathogenesis [49], [50], [51]. CCL24 has many properties comparable with CCL11, and may be able to promote granuloma formation and hepatic fibrosis via a similar mechanism [52]. Thus it is possible that higher eosinophil chemokine expression and eosinophil recruitment contribute to the greater pathology observed in CBA mice. Alternatively, eosinophils may not be directly associated with development of severe pathology, but rather act as indicators of more severe tissue damage.

Expression of AAM markers *(CHI3l3*, *RETNLA*, *MRC1*) was markedly increased in the higher pathology CBA mice compared to BALB/c mice throughout the time course. This may indicate that different macrophage populations constitute the overall hepatic macrophage influx, where macrophages in CBA livers exhibit a more alternatively activated phenotype [53]. AAM have several reported functions including promoting and regulating *S. mansoni*-induced Th2 inflammation and fibrosis [54], [55], [56]. Higher expression of AAM markers in pathology mice CBA in our study suggests that these cells may be involved in promoting pathology in the *S. japonicum* model, however further studies are required to fully dissect the role of these cells.

Fibrosis was induced more rapidly in *S. japonicum*-infected CBA livers than those of BALB/c mice. Although, the fibrotic response of BALB/c mice was similar to CBA mice at 9 weeks p.i., there was an overall trend for lower fibrosis in the former. It would be valuable to observe collagen deposition at later time-points using a larger cohort of mice to assess whether the fibrotic responses are truly similar, or whether CBA mice show a consistently greater degree of fibrosis. Similar variations in the fibrotic response between CBA and BALB/c mice have been reported in pulmonary fibrosis models [57]. Expression of the collagen gene *COL1A1* did not differ between the two mouse strains. Thus, the differences in collagen deposition shown histologically are likely to be attributable to other factors. Known profibrogenic genes, including *PDGF-β*, *TGF-β1* and *CTGF*, were not differentially expressed between BALB/c and CBA mice, indicating that differences in the expression of these profibrotic genes are unlikely to contribute to variations in profibrotic responses between the two mouse strains.

Hepatic stellate cells (HSCs) are the major profibrogenic, collagen producing cells in the schistosome infected liver [13], [58], [59]. The HSC chemokine *CXCL1* and HSC-marker *α-SMA* showed significant differential expression between strains at 4 weeks p.i. The initially lower expression of *CXCL1* and *α-SMA* in BALB/c mice compared to CBA mice may have contributed to the lag in fibrotic development, providing further evidence that early expression of HSC chemokines is critical for the establishment of HSC-associated fibrosis. The similar expression patterns of *CXCL1* and *α-SMA* later in the infection when fibrotic pathology differs between strains suggests that there may be additional factors involved.

The considerably higher expression of fibrosis-associated molecules *MMP12*, *MMP13* and *TIMP1* in CBA mice compared to BALB/c mice, particularly at 7 weeks p.i., coincided with the observed severe fibrotic pathology. MMPs such as MMP13 are involved in cleaving proteins of the extracellular matrix, such as collagen, to remodel tissues [60], [61]. Conversely, MMP12 has recently been identified as a potent inducer of fibrosis during an *S. mansoni* infection, counter-regulating the activity of MMP13 and other matrix metalloproteinases [60]. Up-regulation of *MMP12* in our model may therefore contribute to the higher degree of fibrosis observed in CBA mice. TIMPs subsequently play a role in tissue remodelling and cell proliferation by inhibiting the action of MMPs [13], [62]. The up-regulation of *TIMP1* in the schistosome affected liver may override the collagen-degrading function of the MMPs inhibiting their regulatory function during the fibrotic response. The imbalance between *MMP* and *TIMP* expression has been implicated in the development of fibrosis in the *S. mansoni* murine model [62], but the exact mechanisms by which MMPs and TIMPs promote and/or regulate fibrosis during murine schistosomiasis japonica requires further study.

In summary, we have demonstrated that the mechanisms driving granulomatous pathology during a *S. japonicum* infection are likely to be multi-factorial, with both parasite egg distribution and immune response of a particular mouse strain contributing to the disease outcome. These results provide an important basis for the design of future studies to investigate the precise role of specific genes and/or cells in these processes. Our identification of specific genes and cell types, which likely play key roles in either promoting or regulating the granulomatous response, during schistosomiasis, thereby governing the severity of the resulting disease, may help to guide the future development of novel and effective anti-schistosome interventions.

## Supporting Information

Table S1 **Fold changes and standard deviation of all genes (first 21,986) examined by microarray analysis in BALB/c and CBA mice.** Data is expressed as fold change relative to uninfected controls of the same strain. This gene list has not been filtered for significant signal relative to background or for statistical significance.
(XLS)

Table S2 **Fold changes and standard deviation of all genes (remaining 23,295) examined by microarray analysis in BALB/c and CBA mice.** Data is expressed as fold change relative to uninfected controls of the same strain. This gene list has not been filtered for significant signal relative to background or for statistical significance.
(XLS)

Table S3 **Primers used for real-time PCR validation of microarray expression data.** Primers were designed using Primer-3 software, or sourced from the relevant literature.
(DOC)

Table S4 **Genes showing differing levels of induction between BALB/c and CBA mice following** ***S. japonicum*** **infection.** Data is expressed as fold change relative to uninfected controls of the same strain. Genes were identified by 2-Way ANOVA with Benjamini-Hochberg correction for multiple testing (p≤0.05) of data that had been filtered for significant signal relative to background.
(XLS)

Table S5 **Common functional annotation clusters and significantly associated gene ontologies (GO terms) for up-regulated genes in both BALB/c and CBA mice.**
(DOC)

Table S6 **Common functional annotation clusters and significantly associated gene ontologies (GO terms) for down-regulated genes in both BALB/c and CBA mice.**
(DOC)
