# Supplementary material for: Differential Expression of Chemokine and Matrix Re-Modelling Genes Is Associated with Contrasting Schistosome-Induced Hepatopathology in Murine Models
Source: PLoS Negl Trop Dis. 2011 Jun 7;5(6):e1178. doi: 10.1371/journal.pntd.0001178 (PMC3110159; doi:10.1371/journal.pntd.0001178)
Supplement: Table S3 — Primers used for real-time PCR validation of microarray expression data. Primers were designed using Primer-3 software, or sourced from the relevant literature. (DOC) [file pntd.0001178.s003.doc]

**Table S3. Primers used for real-time PCR validation of microarray expression data.** Primers were designed using Primer-3 software, or sourced from the relevant literature.

| **Gene** | **Forward Primer** | **Reverse Primer** | **Amplicon Length (bp)** | **Source** |
| --- | --- | --- | --- | --- |
| NGP | 5' CCA CTC CGC CTT CTA GTC AG | 5' AGG TCA AGG TCA GGG AGG TT | 186 | Primer-3 |
| MPO | 5' CAC CCT CTT TGT TCG AGA GC | 5' CAA CAC CAA GGG CAG GTA GT | 154 | Primer-3 |
| PPBP | 5' CTG TGC CAA ACC ATT TTC CT | 5' GCC CGT CTT CAT CAT TGT TT | 181 | Primer-3 |
| CTSG | 5' GAG TCC AGA AGG GCT GAG TG | 5' GAT GGC TCT GAG CAC TGT GA | 163 | Primer-3 |
| S100A8 | 5' GGA AAT CAC CAT GCC CTC TAC | 5' GCC ACA CCC ACT TTT ATC ACC | 173 | 5 |
| IL-4 | 5' ACG AGG TCA CAG GAG AAG GGA | 5' AGC CCT ACA GAC GAG CTC ACT C | 101 | 3 |
| IL-13 | 5' GGC AGC ATG GTA TGG AGT GTG | 5' TGG GTC CTG TAG ATG GCA TTG | 101 | 3 |
| CCL24 | 5' TCA CCA AGA AGG GCC ATA AG | 5' TCC AAG TTC AGG GAC AGA GG | 184 | 1 |
| CYP2A4 | 5' GAC CGA ATG AAG ATG CCC TA | 5' TGA AGT CTT TGG GGT TGG AG | 202 | 1 |
| COL1A1 | 5' AAC TGG ACT GTC CCA ACC CC | 5' TCC CTC GAC TCC TAC ATC TTC TG | 100 | 2 |
| HPRT | 5’ GTT GGA TAC AGG CCA GAC TTT GTT G | 5’ GAT TCA ACT TGC GCT CAT CTT AGG C | 163 | 4 |
| 1. Burke, M. L., *et al* (2010). *PLoS Neglected Tropical Diseases*, 4, e598. | | |  |  |
| 2. Sandler, N.G, *et a*l (2003). *The Journal of Immunology*, 171: 3655–3667. | | |  |  |
| 3. Hesse, M, *et al* (2004). *The Journal of Immunology*, 172: 3157-3166 | | |  |  |
| 4. Amante, F.H, *et al* (2007). *The American Journal of Pathology*, 171: 548-549. | | |  |  |
| 5. Rodriguez, A, *et al* (2007). *BMC Genomics*, 8, 379. | |  |  |  |
