# Supplementary material for: Differential Expression of Chemokine and Matrix Re-Modelling Genes Is Associated with Contrasting Schistosome-Induced Hepatopathology in Murine Models
Source: PLoS Negl Trop Dis. 2011 Jun 7;5(6):e1178. doi: 10.1371/journal.pntd.0001178 (PMC3110159; doi:10.1371/journal.pntd.0001178)
Supplement: Table S5 — Common functional annotation clusters and significantly associated gene ontologies (GO terms) for up-regulated genes in both BALB/c and CBA mice. (DOC) [file pntd.0001178.s005.doc]

**Table S5. Common functional annotation clusters and significantly associated gene ontologies (GO terms) for up-regulated genes in both BALB/c and CBA mice.**

| **Annotation Cluster** | **Enrichment Value*** | **Biological Terms** | **P-Value*** |
| --- | --- | --- | --- |
|  |  | GO:0002376~immune system process (176) | 2.85E-41 |
| 1. "Immune | 31.01 | GO:0006955~immune response (119) | 7.17E-33 |
| Response" |  | GO:0050896~response to stimulus (302) | 4.51E-21 |
|  |  | *No other ontologies* |  |
|  |  | GO:0009611~response to wounding (99) | 2.57E-31 |
| 2. "Inflammatory |  | GO:0009605~response to external stimulus (134) | 7.27E-28 |
| and Wound | 25.31 | GO:0006954~inflammatory response (72) | 3.94E-26 |
| Response" |  | GO:0006952~defense response (102) | 6.05E-25 |
|  |  | GO:0006950~response to stress (181) | 6.23E-20 |
|  |  | GO:0040011~locomotion (71) | 3.61E-13 |
| 3. "Chemokine |  | GO:0006935~chemotaxis (34) | 2.05E-12 |
| and Cytokine | 9.84 | GO:0042330~taxis (34) | 3.19E-12 |
| Activity" |  | GO:0008009~chemokine activity (20) | 3.26E-12 |
|  |  | GO:0042379~chemokine receptor binding (20) | 5.91E-12 |
|  |  | GO:0050865~regulation of cell activation (44) | 7.81E-15 |
| 4. "Cell |  | GO:0002694~regulation of leukocyte activation (42) | 6.74E-14 |
| Activity" | 9.64 | GO:0051249~regulation of lymphocyte activation (40) | 1.63E-13 |
|  |  | GO:0050867~positive regulation of cell activation (32) | 9.45E-13 |
|  |  | GO:0050863~regulation of T cell activation (30) | 5.14E-10 |
|  |  | GO:0040011~locomotion (71) | 3.61E-13 |
| 5. "Cell |  | GO:0016477~cell migration (47) | 2.23E-09 |
| Migration and | 7.76 | GO:0006928~cell motion (58) | 1.25E-07 |
| Locomotion" |  | GO:0048870~cell motility (48) | 2.15E-07 |
|  |  | GO:0051674~localization of cell (48) | 3.83E-07 |
|  |  | GO:0001503~ossification (5) | 0.0023176 |
|  |  | GO:0060348~bone development (5) | 0.0032946 |
| 6. "Ossification" | 1.60 | GO:0031214~biomineral formation (3) | 0.0147094 |
|  |  | GO:0001501~skeletal system development (5) | 0.0151607 |
|  |  | GO:0001649~osteoblast differentiation (3) | 0.0246535 |

* Enrichment values and p-values presented were generated from the 7 week p.i. time-point, and were similar in both the 4 and 9 week p.i. time-points. Parentheses represent the number of genes that shared the ontology. Enrichment value represents the negative log transformation of the geometric mean of p-values associated with each biological term in the cluster. This is a measure of confidence that a particular cluster is significant due to a large number of differentially expressed genes.
