# Supplementary material for: Differential Expression of Chemokine and Matrix Re-Modelling Genes Is Associated with Contrasting Schistosome-Induced Hepatopathology in Murine Models
Source: PLoS Negl Trop Dis. 2011 Jun 7;5(6):e1178. doi: 10.1371/journal.pntd.0001178 (PMC3110159; doi:10.1371/journal.pntd.0001178)
Supplement: Table S6 — Common functional annotation clusters and significantly associated gene ontologies (GO terms) for down-regulated genes in both BALB/c and CBA mice. (DOC) [file pntd.0001178.s006.doc]

**Table S6**. Common functional annotation clusters and significantly associated gene ontologies (GO terms) for down-regulated genes in both BALB/c and CBA mice.

| **Annotation Cluster** | **Enrichment Value*** | **Biological Terms** | **P-Value*** |
| --- | --- | --- | --- |
|  |  | GO:0055114~oxidation reduction (115) | 2.25E-54 |
| 1. "Oxido- | 43.42 | GO:0016491~oxidoreductase activity (116) | 3.24E-50 |
| reductase |  | GO:0009055~electron carrier activity (48) | 7.37E-28 |
| Activity" |  | *No other ontologies* |  |
|  |  | GO:0019752~carboxylic acid metabolic process (103) | 2.59E-66 |
| 2. "Metabolic |  | GO:0042180~cellular ketone metabolic process (105) | 4.99E-64 |
| Processes" | 22.95 | GO:0006631~fatty acid metabolic process (46) | 4.98E-31 |
|  |  | GO:0006629~lipid metabolic process (82) | 2.60E-29 |
|  |  | GO:0009308~amine metabolic process (48) | 3.74E-20 |
|  |  | GO:0046394~carboxylic acid biosynthetic process (27) | 2.05E-15 |
| 3. "Biosynthetic |  | GO:0016053~organic acid biosynthetic process (27) | 2.87E-15 |
| Processes" | 8.02 | GO:0008610~lipid biosynthetic process (29) | 2.26E-09 |
|  |  | GO:0006633~fatty acid biosynthetic process (12) | 7.03E-06 |
|  |  | GO:0006694~steroid biosynthetic process (10) | 1.13E-04 |
|  |  | GO:0019842~vitamin binding (26) | 1.31E-13 |
| 4. "Ion and |  | GO:0030170~pyridoxal phosphate binding (14) | 1.82E-08 |
| Vitamin Binding" | 7.11 | GO:0070279~vitamin B6 binding (14) | 1.82E-08 |
|  |  | GO:0009069~serine family amino acid metabolic process (9) | 6.44E-08 |
|  |  | GO:0016769~transferase activity, transferring nitro groups (7) | 6.66E-04 |

* Enrichment values and p-values presented were generated from the 7 week p.i. time-point, and were similar in both the 4 and 9 week p.i. time-points. Parentheses represent the number of genes that shared the ontology. Enrichment value represents the negative log transformation of the geometric mean of p-values associated with each biological term in the cluster. This is a measure of confidence that a particular cluster is significant due to a large number of differentially expressed genes.
